# The use of patient-reported outcome measures in primary care: applications, benefits and challenges

**DOI:** 10.1186/s41687-021-00361-7

**Published:** 2021-10-12

**Authors:** Krista Brower, Margo Schmitt-Boshnick, Michel Haener, Shea Wilks, Allison Soprovich

**Affiliations:** 1 Edmonton Oliver Primary Care Network, #130, 11910 111 Ave, Edmonton, AB Canada; 2 Red Deer Primary Care Network, 5120 47 St, Red Deer, AB Canada; 3 Grande Prairie Primary Care Network, #104, 11745 105 St, Grande Prairie, AB Canada; 4 Palliser Primary Care Network, #104, 140 Maple Ave SE, Medicine Hat, Alberta, Canada; 5 Alberta PROMs and EQ-5D Research and Support Unit (APERSU), 2-040 Li Ka Shing Center for Health Research Innovation, University of Alberta, Edmonton, AB T6G 2E1 Canada

## Abstract

PROMs use in primary care has expanded from simply describing patient populations to contributing to decision-making, in response to the increasingly complex, ever-changing healthcare environment. In Alberta, primary care is organized into primary care networks (PCNs), where family physicians are grouped geographically and supported by allied health professionals. PCNs implement programs and services in response to local population health needs with frequent evaluation, often incorporating PROMs for this purpose. As PCN programs and services vary greatly across Alberta, so do their use of PROMs. An area of commonality is the use of the EQ-5D-5L instrument; 29 out of 41 PCNs are registered and licensed to use the instrument. It is often administrated by paper, pre- and post-program, and in combination with other specific measures, depending on the program or target population. Some PCNs share programming and therefore outcome measurement, but often the selection, implementation (including training and administration procedures) and evaluation/reporting of PROMs are unique to the PCN. As well, data analysis is largely dependent on the size and capacity of the PCN. Using PROMs for PCN program evaluation supports clinical understanding and complements clinical outcomes. PROMs describe the population attending a program, as well as provide an element of consistency when examining trends across multiple programs or timepoints. This contributes to inquiries and decisions around program development, components, administrative features, resource allocation and delivery. Challenges of PROMs use in primary care include the absence of cohesive data capture technology. This limits data capabilities and presents difficulties with data fidelity, storage, export, and analysis. Additionally, this real-world application lacks a control arm and presents methodological challenges for comparative research purposes. Furthermore, capturing long term patient outcomes poses administrative challenges of multiple follow ups. More research is required into best reporting mechanisms to ensure the data is used to its full potential. To overcome these challenges, leadership and clinician engagement are key. As well, determining consistent PCN PROM reporting requirements will ensure data are comparable across PCNs and contribute to provincial level evaluations, further supporting the movement towards overall health system quality improvement.

## Primary care in Alberta

In Alberta, primary care is largely organized into primary care networks (PCNs). PCNs, where member family physicians are grouped geographically, were established in 2003 to enhance primary care services by focusing care in the community and reducing patient involvement with the acute care system [1]. PCNs, in alignment with the Patient’s Medical Home (PMH) model, support member physicians in a variety of ways, including team-based care, electronic medical record (EMR) optimization, panel management, quality improvement expertise, and other functions [2]. PCNs were formed under the notion of “local solutions for local problems”, and offer variable programs and services to meet local population needs. Examples include chronic disease management; pain management clinics; mental health services; and educational classes. Many PCN have formal affiliations with Alberta Health Services (AHS), but PCN are independent organizations led by a board of physicians and programs and services are provided outside of the AHS environment. PCNs are funded by and accountable to Alberta Health, the provincial government, with an accountability framework requiring each PCN to evaluate its local programs and services for outcome achievement [3]. There is also an expectation that each PCN engages in ongoing performance measurement and continuous quality improvement.

There are currently 41 PCNs in Alberta, involving more than 3800 family physician members and more than 1400 other health professionals [4]. Approximately 84% of family physicians in Alberta are members of a PCN [3], with each having a targeted patient panel ranging from approximately 900–2000 [5]. These patients have access to primary care programming provided by allied health professionals, multidisciplinary teams and group health programs, all of which support physicians in patient care. PCNs have been effective in increasing access to primary care services, with evaluation showing a 91% greater attachment to a regular family physician as a function of PCN services, and 73% of physicians reporting that they referred patients to PCN multidisciplinary programming [6]. Ultimately, primary care is set up in localized networks that extend beyond the primary care clinic, which has implications for the implementation of patient-reported outcome measures.

## PROMs in primary care

Historically, patient-reported outcome measures (PROMs) were used in clinical effectiveness research [7], however, primary care has a much broader application than specialty health or acute health [8, 9]. Primary care patients often have divergent health needs and multiple, complex conditions [10].

In this setting, PROMs were traditionally used to inform clinical care for specific health interventions, like screening and management of depressive symptoms [11]. However, we now exist in a modern, increasingly complex, ever-changing health environment that demands improvement in outcomes for patients and sustainability in the health system [12]. This creates an opportunity to incorporate PROMs in other aspects of primary care practice.

**Fig. 1:**
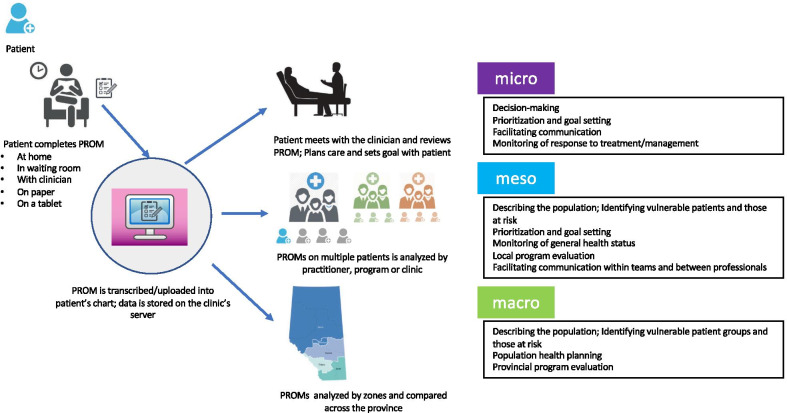
Applications of PROMs in Primary Care Networks at the micro, meso and macro level

The incorporation of PROMs in primary care provides a patient-centric reporting mechanism that can achieve multiple objectives, at micro, meso and macro level applications (Fig. 1). Given that program evaluation is a common meso level application in PCNs, this paper focuses on implementing PROMs in primary care for this purpose.

## PROMs implementation in PCNs

In response to the need for accountability, performance measurement and quality improvement, PROMs were largely implemented in PCNs to capture the patient’s perspective alongside other outcomes. In 2013, a set of common indicators were included in Alberta PCNs’ grant agreements with Alberta Health; the indicator guidance included requiring the EQ-5D be completed and reported as “percentage of patient with chronic condition who report maintaining or improving quality-of-life as measured by the EQ-5D” [3]. However, PCNs vary greatly in their implementation of PROMs, including the selection, administration, data use and reporting of PROMs. For this reason, we outline here only four examples of implementation, and discuss the benefits and challenges broadly (Table 1).

**Table 1:** The application of PROMs in four PCNs

PCN and program	PROMs administered in program and purpose	Frequency of PROMs administration	Frequency of PROMs reporting
Edmonton Oliver PCN—mental health services (short-term 1:1 counselling)	EQ-5D-5L (quality of life)	Initial appointment	Point of care analysis (during initial appointment)
	PHQ-9 (depression)	Follow-up appointment OR 6 months, whichever is first	Annual aggregate reporting
	OQ-45 PROM (psychotherapy progress)		
Grande Prairie PCN—persistent pain program (self-management program)	EQ-5D-5L (quality of life)	Initial appointment	Bi-annual organizational evaluation reporting
	PSEQ PROM (pain self-efficacy)	All appointments with case manager	Annual report to funder
Palliser PCN—behavioural health consultant (BHC) role (staff co-located in PCN health homes)	BHC-7 (functional assessment: patient level; fidelity to BHC model: PCN level)	BHC-7: Each BHC visit	Annual aggregate reporting
	PHQ-9 (depression)	Other PROMs: as required to assess baseline and progress	Occasional reporting to support individual provider improvements in efficiency and effectiveness of patient care
	GAD-7 (anxiety)		
	Burns anxiety inventory store		
Red Deer Primary Care Network—happiness basics 7 week group program (self-management)	SF-12v2 or the EQ-5D-5L (Health Quality of Life)	Administered at week 1 and week 7	Post program reports to program facilitators and leadership team
	Mental Health Continuum-Short Form (emotional, social, and psychological well-being)		Quarterly reports to Board and Physician Community of Practice
	Happiness Thermometer (level of happiness)		Annual reports to Board, funder, staff and community

For PCNs, both condition specific and generic PROMs can be used simultaneously for specific patient populations or conditions (e.g., mental health) or for the general population (e.g., EQ-5D-5L) depending on the purpose or goal of the measurement. Use of PROMs in PCNs has evolved from a singular clinical usage into combinations of PROMs administered to primary care patients with chronic, complex, and comorbid conditions, at multiple timepoints to demonstrate change.

PROMs used in PCNs vary greatly across Alberta. Currently, 29 out of 41 PCNs are registered and licensed to use the EQ-5D, often in combination with a disease or condition specific measure. These 29 PCNs are from a variety of geographical areas, both urban and rural settings, with a varied population size and distribution. Many PROMs are used for a particular program and others for routine outcome measurement across the PCN. Some PCNs share programming and therefore outcome measurement, but often the selection, implementation (including training and administration procedures) and reporting of PROMs are unique to the PCN. Implementation strategies and administration vary across PCNs, depending on the purpose and selected PROM. In program evaluation the majority of PCNs use paper-based tools and measure outcomes pre and post program, although many are shifting to online capture.

The ability for PCNs to use and report PROMs data differs across Alberta, and is based on the size and capacity of the PCN. Although PROMs can be employed at the micro, meso and macro levels in primary care (Fig. 1), many do not use and report data at all three levels. For micro level applications, PROMS are effective tools for driving clinical conversations with patients. Although the benefit to enhancing patient-provider communication has been well documented [13], there is a need for further research on the effects of micro level PROMs applications on direct clinical outcomes in primary care. For macro level applications, comparing to provincial norms requires aggregating data across the PCN patient population and a high degree of analytical capacity. PROMs are well used in research and clinical care; however, their use in primary care and program evaluation in Alberta is new.

## PROMs use for program evaluation in PCNs

Traditional research typically applies rigorous study designs in an attempt to establish causal links between an intervention and improved patient outcomes, such as a PROM as a primary outcome. In program evaluation, as is often applied in the day-to-day practice of primary care, PROMS can be used as one indicator of many that contribute to an understanding of the effectiveness of a program on an annual basis, or in comparing a new program iteration to standard care. Program evaluation focuses not on creating generalizable, comparative information to contribute to the literature, but rather to make local judgements on a program’s success [14], rooted in the unique context of the program, to assist with decision-making.

As a meso level application, PROMs complement and add the patient’s perspective to the traditional focus on clinical attainment at the program level. A key aspect of the PMH model of care includes a greater focus on preventative care and patient self-management [2]. For this reason, many PCN programs target patient behaviour change, such as patient awareness and knowledge of condition and readiness for change. In this context, many metrics are needed to capture the overall impacts of programs; PCN program evaluation often involves using PROMs in conjunction with other measurements to inform comprehensive outcome evaluation of programming. PROMs contribute to the larger picture of performance monitoring and evaluation within PCNs as unique indicators.

In Table 1, we outline four unique examples of Alberta PCN programs tailored to their local population, and their relevant PROMs along with administration and reporting features. Each of these PCN applications underwent extensive internal implementation planning including the selection of the most appropriate PROM(s) for the purpose and target population, reflecting on administration factors, preparing training and engagement activities, as well as plans for analysis and reporting. Although many implementation considerations are shared, the process is iterative and varied. These PCNs have dedicated evaluation staff, thus demonstrating commitment, capacity and scope to implement PROMs. As PROMs are emerging in primary care in Alberta, these PCNs may also be considered ‘early adopters’, signifying their efforts and innovation to explore evolving concepts.

## Benefits of PROMs in program evaluation

Utilizing PROMs to inform program evaluation at a meso level has many benefits for PCNs. The collection of PROMs supports clinical understanding of programs and complements clinical care outcomes. PROMs contribute to program evaluation by tracking program impacts as reported by patients, which has implications for operational effectiveness including resource allocation. Descriptive scores generated from PROMs can be used as a comparison across programs, to inform their components, administrative features and delivery. PROMs can also provide an element of consistency when examining trends across multiple programs or multiple timepoints. Given that the administration of PROMs by PCN clinicians is increasingly becoming part of a routine standard of care, these results can be leveraged to meet funder accountability requirements as well.

PROMs have also contributed to data that can be triangulated with other metrics to describe the performance of the PCN. In this way, PROMs are administered before patients attend a PCN program and afterwards, and that score difference can influence PCN programming. It can demonstrate program benefits, or illustrate the need for further program assessment or development where scores decline in comparison to other programs or populations. While ideal, this type of data capture is not typical of current primary care settings and varies considerably across PCNs.

## Challenges using PROMs in program evaluation

Challenges to use PROMs in PCN program evaluation persist. The administration of PROMs in primary care can be more difficult than in acute care settings, where all providers utilize cohesive technology system to house all their data. Primary care has lagged in many technological advancements and not all PCN locations have EMRs or other compatible databases that house patient data. For those that do, the administration of the PROMs through a clinical workflow can be complex, with multiple opportunities for error. Additionally, it is the PCN that coordinates, negotiates and ultimately pays for EMR systems and their data collection and storage, in which PROMs are not easily separated from other outcomes and functions. Indeed, the lack of consolidated and compatible EMR systems in primary care in Alberta has been previously documented [15, 16], and contributes to data analysis difficulties.

PCNs also vary in terms of data storage and analytical capacity. There are often barriers to efficiently displaying the information for clinicians to both access and use PROM(s) in clinical conversations, while simultaneously storing the data in a standardized electronic format. Maintaining adherence to the data quality specifications and implementation practices is complicated by the reality that PCN programs are often delivered at multiple clinical sites separate from information management and evaluation staff. As well, many patients attend PCN programs for short-term interventions, reducing the ability to implement and follow-up on an intervention, which is challenging for longitudinal data collection to measure longer term outcomes.

To overcome these challenges, leadership and clinician engagement is paramount. Once staff members see the value of PROMs, the administration, collection, workflow integration, infrastructure, and analyses will facilitate implementation [3]. Increased support to optimize use of clinic EMR systems in administrating, charting, sharing and setting re-assessment intervals for PROMs can ensure collection efficiency and thus potential value for evaluation. Further, it is important in program evaluation to utilize PROMs as part of a strong evaluation framework with multiple data sources to accurately assess the impact of the program for the patient (e.g., patient experience surveys, attendance rates, clinical experience, PROMs). Additionally, determining consistent PROM reporting requirements in primary care will ensure data are comparable across PCNs and will contribute to provincial level evaluations, further supporting the movement towards overall health system quality improvement.

## Conclusion

Although PROMs provide a base of consistent, methodologically sound information directly reported by patients, their implementation and use to describe the achievements of programs and services can be challenging. In Alberta, some PCNs have innovatively used PROMs along with other data sources, for the comprehensive evaluation of local primary care programs and services. Here we described the PCNs use at the meso level application in program evaluation (Fig. 1) which in turn may drive further PROM use in clinical care and research in primary care. Future directions may include in-depth examinations on the implementation across PCNs, including engagement activities of patients and clinicians, identifying priorities, and informing decision-making.

## Data Availability

Not applicable.

## Abbreviations

AHS: Alberta health services
BHC: Behavioural health consultant
BHC-7: Behavioural health consultant 7-item PROM
EMR: Electronic medical record
EQ-5D: EuroQol 5 dimensions
GAD -7: General anxiety disorder 7
PCN: Primary care network
PHQ-9: Patient health questionnaire 9
PMH: Patient medical home
PRO: Patient reported outcome
PROM: Patient reported outcome measure
PSEQ: Pain self-efficacy questionnaire
OQ-45: Outcome questionnaire 45
SF-12: Short form 12
